# Decreased Connection Between Reward Systems and Paralimbic Cortex in Depressive Patients

**DOI:** 10.3389/fnins.2018.00462

**Published:** 2018-07-09

**Authors:** Tongjian Bai, Meidan Zu, Yang Chen, Wen Xie, Chunlan Cai, Qiang Wei, Gong-Jun Ji, Yanghua Tian, Kai Wang

**Affiliations:** ^1^Department of Neurology, The First Affiliated Hospital, Anhui Medical University, Hefei, China; ^2^Anhui Mental Health Center, Hefei, China; ^3^Anhui Province Key Laboratory of Cognition and Neuropsychiatric Disorders, Hefei, China; ^4^Department of Medical Psychology, The First Affiliated Hospital, Anhui Medical University, Hefei, China; ^5^Collaborative Innovation Center of Neuropsychiatric Disorders and Mental Health, Hefei, China

**Keywords:** depression, reward system, orbitofrontal cortex, nucleus accumbens, temporal pole

## Abstract

Despite decades of research on depression, the underlying pathophysiology of depression remains incompletely understood. Emerging evidence from task-based studies suggests that the abnormal reward-related processing contribute to the development of depression. It is unclear about the function pattern of reward-related circuit during resting state in depressive patients. In present study, seed-based functional connectivity was used to evaluate the functional pattern of reward-related circuit during resting state. Selected seeds were two key nodes in reward processing, medial orbitofrontal cortex (mOFC) and nucleus accumbens (NAcc). Fifty depressive patients and 57 healthy participants were included in present study. Clinical severity of participants was assessed with Hamilton depression scale and Hamilton anxiety scale. We found that compared with healthy participants, depressive patients showed decreased connectivity of right mOFC with left temporal pole (TP_L), right insula extending to superior temporal gyrus (INS_R/STG) and increased connectivity of right mOFC with left precuneus. Similarly, decreased connectivity of left mOFC with TP_L and increased connectivity with cuneus were found in depressive patients. There is also decreased connectivity of right NAcc with bilateral temporal pole, as well as decreased connectivity of left NAcc with INS_R/STG. In addition, the functional connectivity of right nucleus accumbens with right temporal pole (TP_R) was negatively correlated with clinical severity. Our results emphasize the role of communication deficits between reward systems and paralimbic cortex in the pathophysiology of depression.

## Introduction

Depression is among common psychiatric disorders and the leading causes of disability worldwide (Ferrari et al., 2013). Despite decades of research on depression, the pathological neural mechanisms of depression remains incompletely understood. As a heterogenous psychiatric disorder, diverse symptoms of depression may attribute to distinct pathophysiology (Drysdale et al., 2016; Guo et al., 2016). Increasingly, anhedonia is regarded as a cardinal feature of depression (Pizzagalli, 2014) and associated with increased risk for suicide (Ducasse et al., 2018) and poor treatment outcome (Vrieze et al., 2014). Anhedonia is defined as reduced interest or pleasure previously rewarding activities, part of a spectrum of reward circuit abnormalities (Der-Avakian and Markou, 2012). Convergent studies implicated the dysfunction of reward brain system in the neurobiology of anhedonia (Auerbach et al., 2017; Karcher et al., 2017). Indeed, behavioral studies have revealed the abnormality during reward-related processing in depression (Rizvi et al., 2018), displayed several types such as reward response bias, impaired reward learning ability and increased risk avoidance (Smoski et al., 2009).

The orbitofrontal cortex (OFC) is a key node in processing salience and magnitude of rewards (O’Doherty et al., 2001; Gottfried et al., 2003; Schoenbaum et al., 2011). Besides, OFC also plays a critical role in integrating reward information based on its strong anatomical connection with reward-related regions sensory, limbic and ventral striatal cortex (Kahnt et al., 2012). Ventral striatum is another core region in reward-related processing (Bartra et al., 2013; Sescousse et al., 2013). As a part of the ventral striatum, the nucleus accumbens (NAcc) is an important component of the reward circuit in the brain (Misaki et al., 2016), mainly responsible for mediating hedonic perception of rewards. In addition to perception of rewards, NAcc also takes on as a modulator in motivation-related behavior, which can influence several symptoms in depression, such as lack of motivation, anergia, or psychomotor slowing (Salamone et al., 2005).

Indeed, evidence from task-based neuroimaging studies has validated the maladaptive neural response of OFC and NAcc in reward-related processing in patients with depression. Blunted activity in OFC and NAcc for reward outcomes was consistently identified in depression (Tremblay et al., 2005; Pizzagalli et al., 2009). Depressive patients also exhibit hypoactivation of medial OFC in the neural coding for reward prediction (Rothkirch et al., 2017), as well as the decreased activity in NAcc (Ubl et al., 2015). In addition, the aberrancy of OFC and NAcc in depression is also supported by evidence from structural findings (Kempton et al., 2011; Lu et al., 2017).

Besides task-based and structural methods, another functional neuroimaging tool, resting-state functional magnetic resonance imaging (RS-fMRI), enables the detection of spontaneous brain activity to identify brain dysfunction in diseases (Biswal et al., 1995; Zhang and Raichle, 2010; Ji et al., 2017). With the non-invasive and task-independent feature, RS-fMRI has been increasingly applied in several mental diseases, especially in depressive disorder (Jalbrzikowski et al., 2017; Anhoj et al., 2018; Chen et al., 2018). Dysfunction within reward circuit among depressive individuals has been recognized using RS-fMRI (Cheng et al., 2016; Gong et al., 2017). Seed-based functional connectivity is one of technique to measure multi-regional cooperation within a special network. With the seed of medial and lateral OFC, Cheng et al. (2016) found that depressive patients exhibit reduced functional connectivity of medial OFC (mOFC) with temporal gyrus but increased functional connectivity of lateral OFC with precuneus and angular gyrus. Accordingly, they concluded medial reward and lateral non-reward orbitofrontal cortex circuits in depression. Consistent with this view, Gong et al. (2017) found decreased connectivity of NAcc with OFC, dorsomedial prefrontal cortex, superior temporal gyrus and insular lobe in depression. However, there are also studies reported against this view (Avery et al., 2014; Rzepa and McCabe, 2016). Diversity of seeds selection in different studies may explain these discrepancies. There is few work used diverse seeds within reward network in a single study to validate the reward-network abnormality in depression.

In present study, we investigated the functional coupling of depressive patients within reward network with diverse seeds (mOFC and NAcc). We hypothesized that there are similar alteration of coupling pattern between the NAcc-based and mOFC-based reward circuit in depressive individuals. In addition, we also investigated the neural alteration of reward-circuits coupling and clinical severity.

## Materials and Methods

### Participants

Fifty patients with depressive episode from Anhui Mental Health Center were included in present study. All patients diagnosed with depressive episode according to the Diagnostic and Statistical Manual of Mental Disorders, Fourth Edition. Patients were excluded if they met the following exclusion criteria: (1) a history of ECT in the last 3 months; (2) age > 65 years; (3) diagnosed with substance misuse, schizoaffective disorder, or schizophrenia; (4) past or current neurological illness; (5) head motion exceeding 2 mm in translation or 2° in rotation during fMRI scanning; (6) other contraindications of MRI scan. Clinical severity of patients was assessed with Hamilton depression scale (HAMD) and Hamilton anxiety scale (HAMA). We also recruited 57 healthy participants who met the same exclusion criteria as depressive patients except the diagnosis of depressive disorder. This study was carried out in accordance with the recommendations of Human Brain Imaging Collection, Anhui Medical University Ethics Committee. The protocol was approved by the Anhui Medical University Ethics Committee. All subjects gave written informed consent in accordance with the Declaration of Helsinki.

### MRI Data Acquisition

Resting-state and structural images of participants were acquired at the First Affiliated Hospital of Anhui Medical University. Participants were instructed to keep their eyes closed and move and think as little as possible during the MRI scanning. Resting-state MRI scans were conducted under a 3.0 T MRI scanner (Signa HDxt 3.0 T, GE Healthcare, Buckinghamshire, United Kingdom) composed of 240 echo-planar imaging volumes with the following parameters: TR = 2000 ms; TE = 22.5 ms; flip angle = 30°; matrix size = 64 × 64, field of view = 220 mm × 220 mm; slice thickness = 4 mm; 33 continuous slices (one voxel = 3.4 mm × 3.4 mm × 4.6 mm). High resolution three-dimensional brain volume imaging (3D BRAVO) for each participant was also acquired as an anatomical reference with following parameters: TR = 8.676 ms; TE = 3.184 ms; inversion time = 800 ms; flip angle = 8°; field of view = 256 mm × 256 mm; slice thickness = 1 mm; voxel size = 1 mm × 1 mm × 1 mm.

### Functional Data Preprocessing

Functional data pre-processing was conducted with the Data Processing Assistant for Resting-State Functional MR Imaging toolkit (DPARSF), a software package based on Statistical Parametric Mapping software (SPM8^1^) and Resting State Functional MR Imaging Toolkit (REST^2^). The first 10 volumes were discarded to exclude the influence of unstable longitudinal magnetization. The remaining volumes were processed using the following steps: slice timing correction; realignment; coregistering to respective structural images; smoothed with a Gaussian kernel of 6 mm × 6 mm × 6 mm. The resulting images were regressed out nuisance signals, including global mean, white matter, cerebrospinal fluid signals, and 24 head-motion parameters. Finally, the images were filtered with a temporal band-pass of 0.01–0.1 Hz.

### Functional Connectivity of Reward Circuits

**FIGURE 1:**
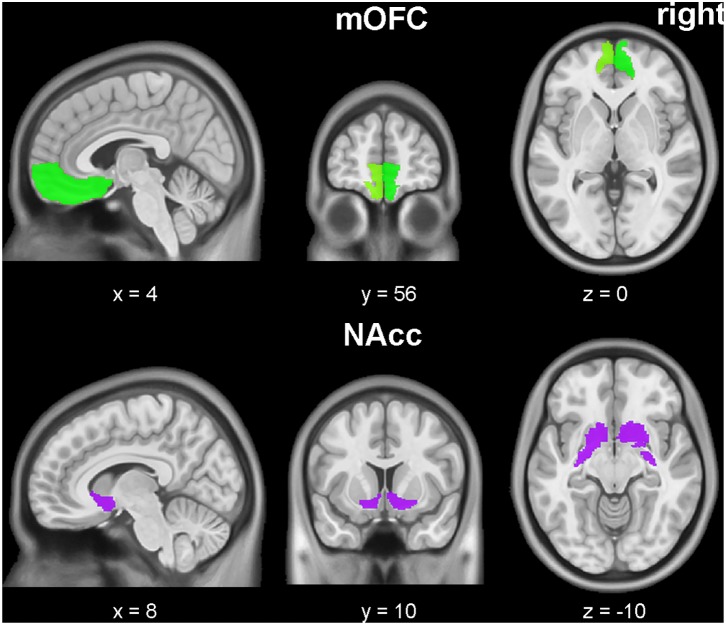
Medial orbitofrontal cortex (mOFC) (right and left, green) and nucleus accumbens (NAcc) (right and left, purple) seed ROIs. The underlying brain template is from MNI152 standard-space structural image. The x, y, z values are in MNI coordinates.

The bilateral masks of NAcc and mOFC were defined according to the Human Brainnetome Atlas (Fan et al., 2016), a new brain atlas build upon connectional architecture. The mOFC masks consist of three subregions, labeled as ID 41, 47, 49 (left mOFC) and ID 42, 48, 50 (right mOFC) in Human Brainnetome Atlas. All seeds were showed in **Figure 1**. For each seeds, the functional connectivity was acquired by Pearson correlation coefficients between the mean time series of seed region and all brain voxels (defined by the binary gray matter mask in SPM). A Fisher’s Z transformation was applied to improve the normality of correlation coefficient values. Finally, two-sample *t*-tests were applied to map group difference of connectivity map for each seed between depressive and healthy participants.

### Statistical Analysis

For group-level structural and functional connectivity analyses, we performed two-sample *t*-tests for comparisons between the depressive patients and healthy controls for connectivity map of each seed. All statistical maps were thresholded using the Gaussian random field (GRF) correction with a voxel-level threshold of *P* < 0.001 and a cluster-level threshold of *P* < 0.05. We also compared changes in functional connectivity with changes in clinical symptoms among depressed individual using SPSS. The statistical level with *P* < 0.05 was considered as significant of correlation analysis with no correction.

## Results

### Demographic and Clinical Characteristic

Present study included 50 patients with current depressive episode and 57 healthy controls. Demographic characteristic of the two groups are shown in **Table 1**. There was no significant difference between two groups in terms of age or gender. Compared with the healthy controls, depressive individuals presented greater HAMD and HAMA scores.

**Table 1:** Demographic and clinical characteristic.

	Depressive individuals	Healthy controls	*t* or χ^2^	*P*-value
Age (mean ±*SD*)	38.68 ± 11.33	36.68 ± 8.76	1.03^a^	0.31
Gender (M/F)	17/33	22/35	0.24^b^	0.62
HAMD (mean ±*SD*)	22.78 ± 3.96	2.93 ± 1.51	35.10^a^	<0.001
HAMA (mean ±*SD*)	15.10 ± 6.81	2.26 ± 1.34	33.42^a^	<0.001


*^a^t-Value; ^b^chi-square; HAMD, Hamilton depression scale; HAMA, Hamilton anxiety scale; SD, standard deviation; M, male; F, female.*

### Group-Level Comparison of Functional Connectivity of the Bilateral mOFC

**FIGURE 2:**
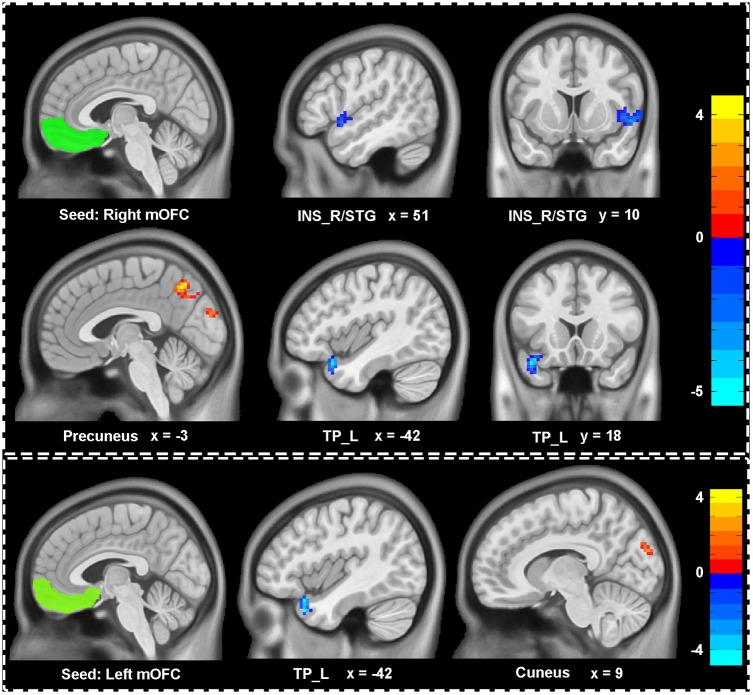
Aberrant functional connectivity of mOFC in depressive patients compared with healthy controls. Depressive patients showed decreased connectivity of right mOFC with left temporal pole (TP_L), right insula extending to superior temporal gyrus (INS_R/STG) and increased connectivity of right mOFC with left precuneus. There were also decreased connectivity of left mOFC with LTP and increased with cuneus. All statistical maps were corrected with Gaussian random field (GRF) method at threshold of voxel *P* < 0.001, cluster *P* < 0.05. The *t* score bars are shown at right. The x, y values are in MNI coordinates.

We explored the difference of functional connectivity between the two groups based on the seed of bilateral mOFC (shown in **Figure 2** and **Table 2**). Compared with healthy controls, depressive individuals presented decreased connectivity of right mOFC with left temporal pole (TP_L) and right insula extending to superior temporal gyrus (INS_R/STG), as well as increased connectivity with left precuneus. For the left mOFC, depressive individuals presented decreased connectivity in TP_L and increased connectivity in right cuneus.

**Table 2:** Brain regions showing significant difference of functional connectivity with the reward network.

Seeds	Abnormal regions	Number of voxels	Peak intensity	Peak coordinates (x, y, z)^a^
Right mOFC	TP_L	45	-5.47	-42, 18, -24
	INS_R/STG	80	-4.46	54, 9, -6
	Precuneus	161	4.65	-3, -63, 48
Left mOFC	TP_L	50	-4.83	-42, 18, -33
	Cuneus	63	4.11	6, -87, 24
Right NAcc	TP_L	36	-4.59	-42, 18, -33
	TP_R	32	-4.38	33, 6, -36
Left NAcc	INS_R/STG	35	-4.36	33, 6, -6


*^*a*^The x, y, and z values are in Montreal Neurological Institute (MNI) coordinates. mOFC, medial orbitofrontal cortex; NAcc, nucleus accumbens; TP_L, left temporal pole; INS_R/STG, right insula extending to superior temporal gyrus; TP_R, right temporal pole.*

### Group-Level Comparison of Functional Connectivity of the Bilateral NAcc

**FIGURE 3:**
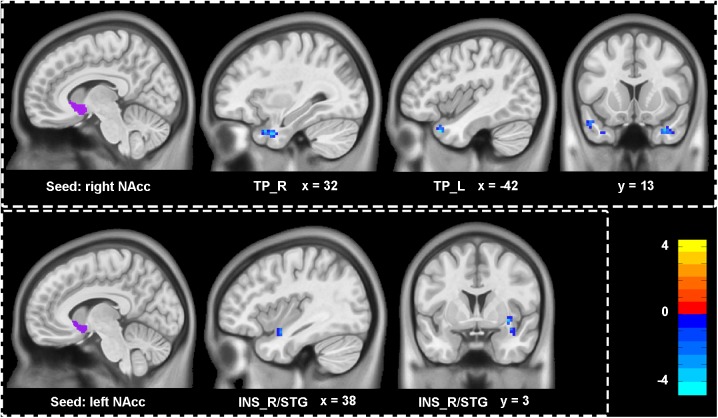
Aberrant functional connectivity of NAcc in depressive patients compared with healthy controls. Depressive patients showed decreased connectivity of right NAcc with left and right temporal pole (TP_L, TP_R), as well as decreased connectivity of left NAcc with right insula extending to superior temporal gyrus (INS_R/STG). All statistical maps were corrected with GRF method at threshold of voxel *P* < 0.001, cluster *P* < 0.05. The *t* score bars are shown at right. The x, y values are in MNI coordinates.

**Figure 3** and **Table 2** showed the difference of functional connectivity based on bilateral NAcc between depressive individuals and healthy controls. Compared with healthy controls, depressive individuals presented decreased connectivity of right NAcc with right temporal pole (TP_R) and TP_L. There was also decreased connectivity of left NAcc with INS_R/STG.

### The Relationship Between Functional Connectivity and the Clinical Severity of Depressive Individuals

**FIGURE 4:**
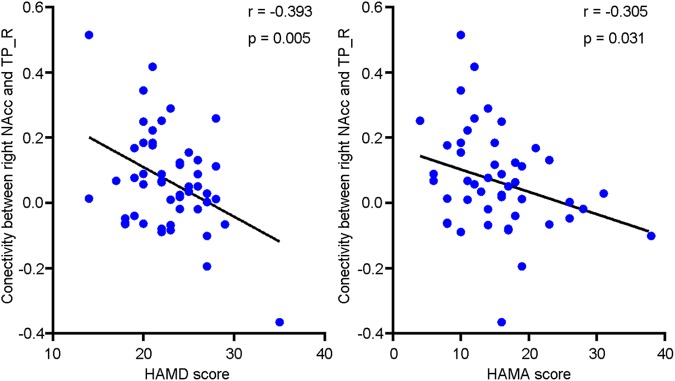
Scatter plots of the negative relationship between clinical severity and functional connectivity of right NAcc with right temporal pole (TP_R). The clinical severity were assessed with Hamilton depression scale (HAMD) and Hamilton anxiety scale (HAMA).

A negative relationship (*r* = -0.393, *P* = 0.005) existed between right NAcc-TP_R connectivity and depressive symptomatology among depressive individuals as shown in **Figure 4**. There was also a negative relationship (*r* = -0.305, *P* = 0.031) between right NAcc-TP_R connectivity and anxiety severity among depressive individuals.

## Discussion

In the present study, we investigated the function-pattern alterations of reward-related circuit during resting state in depressive patients with two key nodes in reward network, mOFC and NAcc. Compared with healthy participants, with both seeds of mOFC and NAcc, depressive individuals displayed decreased connectivity of reward network with paralimbic cortex, including temporal pole (TP), insula, and superior temporal gyrus. Furthermore, decreased connectivity between reward network and paralimbic cortex was significantly associated with clinical severity in depressive individuals.

Consistent with previous task-related neuroimaging studies, our findings with RS-fMRI also revealed the disrupted reward circuits in depressive individuals, specially, a reduction on the connectivity of mOFC and NAcc with paralimbic cortex. It is well-established that mOFC and NAcc play a crucial role in the representation of value-based information (Bartra et al., 2013). Blunt neural responses of mOFC and NAcc toward reward-related stimulus were frequently reported in depressive individuals (Tremblay et al., 2005; Redlich et al., 2015). Reduced activities of mOFC and NAcc during reward processing are associated with clinical characteristic in depression, such as depressive severity, anhedonia severity, and suicide in depression (Misaki et al., 2016; Kim et al., 2017; Rothkirch et al., 2017). Recently, findings from resting-state studies also validated the abnormal coupling of mOFC or NAcc with other brain regions in depressive individuals (Baeken et al., 2017; Gong et al., 2017). Gong et al. (2017) found decreased connectivity of NAcc with OFC, dorsomedial prefrontal cortex, superior temporal gyrus, and insular lobe in depression. Similarly, another study with brain-wide voxel-level resting state neuroimaging analysis revealed reduced functional connectivity of mOFC with temporal gyrus in depression (Cheng et al., 2016). In line with previous studies, based on both the seeds of mOFC and NAcc, we found reduced connectivity of reward system with paralimbic cortex (temporal pole and insular lobe) in depression. Significantly, the connectivity between mOFC and TP is negatively associated with clinical severity.

The TP is a node of paralimbic system with strong connectivity with orbitofrontal cortex, striatum, insula, amygdala, and other emotion-related regions (Fan et al., 2014). As an association cortex, the TP enable multisensory integration and plays key roles in cognitive and socioemotional processing, such as memory, face processing and theory of mind (Olson et al., 2007). The disturbance of these processing is constantly correlated with depression (Zobel et al., 2010; Bistricky et al., 2011). Structural and functional abnormities of the TP have been detected in depression (Beauregard et al., 2006; Peng et al., 2011). Coupled with our findings, decreased mOFC-TP connectivity in depressive individuals has been demonstrated by prior works (Cheng et al., 2016; Rolls et al., 2018). Cheng et al. (2016) concluded that reduced functional connectivity between brain areas involved in pleasant feelings and rewards with memory systems, and that this may be part of the mechanism of depression. This hypothesis is strengthened by the significant relation between the depressive severity and the reduced connection between the mOFC and temporal lobe, revealed by both present and previous studies.

Along with the TP, the insula is another part of paralimbic system and involved in the evaluation of emotional or motivational salience of external and internal stimuli (Damasio et al., 2013). Specially, the insula participate in the representation of subjective value and act as a modulator for neural responses to losses and gains, which contribute to computing the costs and benefits in mixed valence scenarios (Bartra et al., 2013). Abnormal neural responses in insula during reward-related processes were closely related with depressive symptoms (Engelmann et al., 2017). Further, our study found impaired connection between insula and acknowledged reward-related regions, OFC and ventral striatum, in depressive individuals. Indeed, there are strong structural connectivity between insula and acknowledged reward-related regions (Leong et al., 2016; Ghaziri et al., 2017). In line with our finding, previous neuroimaging study suggested that high risk for depression is related with the abnormal connection between OFC and insula during reward-related task (DelDonno et al., 2017). Based on the crucial role of insula in representation of emotional awareness and interoceptive signals, this connectional abnormity was interpreted that the impaired integration of the value of loss with the emotional and interoceptive awareness is correlated with the occurrence of depression (Rolls, 2016).

It is worthy of note that, besides decreased connectivity, increased connection of reward network among depressive individuals was also found within precuneus and cuneus. The precuneus is a key node of default mode network and involved in the sense of self and agency (Cavanna and Trimble, 2006). Increased functional connectivity during reward task between reward network and precuneus has been reported among patients with reward-related disease (Weiland et al., 2013). Studies with resting-state tool found the enhanced connectivity between precuneus and lateral OFC (defined as a non-reward/punishment system in depression) (Cheng et al., 2016). Along with precuneus, the cuneus is another prominent functional hub in the neural model of depression (Tomasi and Volkow, 2011). The cuneus is involved in the perception of facial emotion (Fusar-Poli et al., 2009), which is important for social interaction. Resting neuroimaging study in depression has suggested increased connectivity of reward network with the cuneus and that the enhanced connection was correlated with increased anhedonia severity (Yang et al., 2017). It is suggested be related to the explicit affectively negative sense of the self and increased anhedonia in depression (Rolls, 2016).

It is generally considered that there is high rate of suicide in patients with depression. Patients with suicidal behavior or ideation also presented aberrant reward processing and responses (Dombrovski et al., 2013; Silvers et al., 2016). The decreased connection between reward network and paralimbic cortex in depression may also attribute to the effect of suicide. Besides suicide, as a heterogeneous disease, depression manifest as diverse symptoms, such as anhedonia, low mood, anxiety, and somatic complaints. These symptoms have been associated with impaired reward-related processing (Avinun et al., 2017; Porreca and Navratilova, 2017; Nelson et al., 2018). For example, the increased anxiety symptoms were associated with decreased cortical activity while reward processing in both healthy participants and depressive patients (Nelson et al., 2018). Consistent with this finding, our results also implied the significant correlation between NAcc-TP connection and anxiety severity. In addition, different symptoms may have distinct effects on reward-based processing (Harle et al., 2017). Unfortunately, our study did not include the information about the specific symptoms of depressed patients. The absence of these informations restrains our attendance to understand what particular aspects of depression are associated with abnormal reward-network function. Further examinations are needed to explore the effect of specific symptoms on the reward network in depression.

It is must be acknowledged that there are several additional limitations in our study. On one hand, patients included in current study were given antidepressive medications. Future studies with drug-naïve patients to exclude the effects of medication are necessary. On the other hand, depressive patients enrolled into our study consisted of patients with both unipolar and bipolar depression. Given the different neural pattern of reward networks between unipolar and bipolar depression (Redlich et al., 2015), our findings mixed in factor of diagnostic types. Hence, our results should be interpreted with caution, and future investigations are needed divide participants into subgroups to clarify the distinct mechanisms underlying the specific subtypes of depressive individuals.

## Conclusion

It compared with healthy participants, the depressive individuals showed decreased connectivity of reward network with paralimbic cortex, including TP, insula. The findings were validated with two key seeds of reward network, mOFC and NAcc. Significantly, the decreased connectivity between mOFC and TP was associated with depressive severity. Our study demonstrated reward-network abnormalities among depressive patients in resting-state functional pattern that underlies the pathogenesis of depression. These findings might also imply a potential biomarker for clinical applications.

## Author Contributions

TB and MZ performed the analysis and wrote the paper. YC, WX, CC, and QW helped to collect behavioral and imaging data. G-JJ help for the analysis of resting-state imaging data. YT and KW designed and supervised the present study.

## Conflict of Interest Statement

The authors declare that the research was conducted in the absence of any commercial or financial relationships that could be construed as a potential conflict of interest.

## Footnotes

1 www.fil.ion.ucl.ac.uk/spm

2 http://www.restfmri.net
